# Machine learning extracts marks of thiamine’s role in cold acclimation in the transcriptome of *Vitis vinifera*

**DOI:** 10.3389/fpls.2023.1303542

**Published:** 2023-12-06

**Authors:** Tomas Konecny, Maria Nikoghosyan, Hans Binder

**Affiliations:** ^1^ Armenian Bioinformatics Institute, Yerevan, Armenia; ^2^ Interdisciplinary Centre for Bioinformatics, University of Leipzig, Leipzig, Germany; ^3^ Bioinformatics Group, Institute of Molecular Biology Institute of National Academy of Sciences RA, Yerevan, Armenia

**Keywords:** Self organizing maps, grapevine, temperature stress, vitamin B1, epigenetics, climate change

## Abstract

**Introduction:**

The escalating challenge of climate change has underscored the critical need to understand cold defense mechanisms in cultivated grapevine *Vitis vinifera*. Temperature variations can affect the growth and overall health of vine.

**Methods:**

We used Self Organizing Maps machine learning method to analyze gene expression data from leaves of five *Vitis vinifera* cultivars each treated by four different temperature conditions. The algorithm generated sample-specific “portraits” of the normalized gene expression data, revealing distinct patterns related to the temperature conditions applied.

**Results:**

Our analysis unveiled a connection with vitamin B1 (thiamine) biosynthesis, suggesting a link between temperature regulation and thiamine metabolism, in agreement with thiamine related stress response established in Arabidopsis before. Furthermore, we found that epigenetic mechanisms play a crucial role in regulating the expression of stress-responsive genes at low temperatures in grapevines.

**Discussion:**

Application of Self Organizing Maps portrayal to vine transcriptomics identified modules of coregulated genes triggered under cold stress. Our machine learning approach provides a promising option for transcriptomics studies in plants.

## 1 Introduction

*Vitis vinifera* is a species of grapevine plant that has been cultivated for thousands of years. Its fruit is used both in the production of wine and as a table grape. It is one of the most economically important fruit crops globally. Of the thousands of varieties of grapes, only a few are of commercial significance for wine and table grape production. The European grapevine is adapted to various climatic conditions, but its performance is best in temperate regions with mild winters and warm summers. The greatest challenge that climate change brings to winemaking is unpredictability. Producers used to know which varieties to grow, how to grow them, when to harvest the berries and how to ferment them to produce a consistent, quality wine – but today, every step is subject to uncertainty. In response to these challenges, winemakers are finding ways to preserve traditional as well as economically beneficial grape varieties and their unique qualities under the shifting conditions of global warming, and researchers are integrating knowledge, resources, and services regarding the grapevine (Navarro-Payá et al., 2022).

Temperature studies in vine are crucial as they help understand how variations in temperature affect vine development (de Rességuier et al., 2020; Merrill et al., 2020). Changes in temperature trigger shifts of ripening, which affect grape composition (Leeuwen and Darriet, 2016). For instance, higher temperatures have been found to correlate with flower abortion, potentially leading to decreased wine grape yields (Merrill et al., 2020). Another major challenge faced by grape growers is cold temperature damage, especially during the winter season. In many regions, grapevines are exposed to extreme cold, which can result in injury or death of the plants. Cold tolerance is a critical trait essential for the survival and productivity of grapevines in cold regions. However, the plant resistance to very cold and freezing temperatures has not been extensively studied yet despite recent advances in sequencing and molecular biotechnology (Ren et al., 2023). Also, not much is known about the impact of different temperatures on the gene expression patterns of *Vitis vinifera* (Han et al., 2023). Experiments done under various cold stress conditions showed that the freeze-shock damages plant leaves more than long-term freezing (Londo et al., 2018). The researchers found that the freeze-shock stress limits the sustainability and productivity of grapevines. The transcriptional landscape contrasts observed between low temperature and freezing stresses demonstrate quite different activation of candidate pathways impacting grapevine cold response. Genes from the ethylene signaling, abscisic acid signaling, the *AP2*, *WRKY*, and *NAC* transcription factor families, and starch/sucrose/galactose pathways were among the most observed to be differentially regulated. In response to cold stress, plants possess activation of specific metabolic pathways (including sugar accumulation and biosynthesis of prolines), changes in cell membrane rigidity, activation of calcium signaling and several ice-responding genes (like *ICE1* and *CBFs*), and epigenetic regulation, to protect cells from ice nucleation, control cell membrane stability, scavenge reactive oxygen species, and adapt to cold stress, respectively (reviewed in Theocharis et al., 2012; Satyakam et al., 2022).

In Arabidopsis, the transcription factors AP2/ERF, WRKY, NAC, and MYB are known to enhance plant response to cold stress through various signaling pathways (reviewed in Abdullah et al., 2022). They are involved in the regulation of cold-responsive genes and contribute to increased cold tolerance. Specifically, MYB transcription factors are crucial in cold response due to their influence in regulation of cold-responsive genes, like CBF genes (Wang et al., 2023). Overexpression of certain MYB transcription factors can lead to enhanced tolerance to abiotic stress. Although the specific functions of these transcription factors in the cold acclimation of Arabidopsis are well-studied, there is only limited knowledge about their exact role in cold acclimation of grapevine.

Epigenetic modifications, such as DNA methylation and post-translational modifications of histone proteins, play a crucial role in plant responses to cold stress (Sharma et al., 2022; Rehman et al., 2023). It is widely accepted that grapevine DNA methylation variability is primarily influenced by genotype. However, recent findings suggest that the environment where the grapevine grows can also significantly alter the methylome (Xie et al., 2017; Baránková et al., 2021). Epigenetic mechanisms are also associated with the regulation of metabolite biosynthesis and the accumulation of phenolic compounds in grapevines (He et al., 2010; Marfil et al., 2019). Thiamine, or vitamin B1, acts as a cofactor for several enzymes involved in metabolic pathways. It is crucial for plant health, particularly in defending against pathogens (Subki et al., 2018) and its role is known also in wine production (Bataillon et al., 1996). Thiamine has been found to boost the immunity and defense system of plants, playing a key role in their protection against biotic and abiotic stresses (Subki et al., 2018; Jaiwal et al., 2019). The exposure to abiotic stresses in the plant model organism *Arabidopsis thaliana* results in an upregulation of thiamin biosynthetic gene expression and the thiamine accumulation leading to enhanced tolerance to oxidative stress (Tunc-Ozdemir et al., 2009). However, the relationship between thiamine metabolism and temperature control in grapevines is not known. Our study aims to elucidate the expression of thiamin-biosynthetic genes in grapevines during cold acclimation and freezing stress.

Moreover, transcriptome-wide gene expression studies on larger sample sets are challenging because they aim at extracting relevant biological information such as affected pathways and related marker genes from a multidimensional data landscape with a co-variance structure much more complex than simple case-control settings. A series of machine-learning based methods such as weighted correlation network analysis (WGCNA) (Langfelder and Horvath, 2008) or non-negative matrix factorization (NMF) (Frigyesi and Höglund, 2008) have been developed to solve the problem via appropriate dimension reduction. Self-Organizing Maps (SOM) provide another option for knowledge mining in complex data to extract hidden covariance relations of reduced dimensionality.

The SOM machine learning method was developed by Kohonen over 30 years ago (Kohonen, 1982). It provides a very effective clustering algorithm which can be adapted to a wide range of applications (Loeffler-Wirth et al., 2020). We here make use of the “omics-portrayal” variant of SOM which combines supervised clustering of gene expression profiles into a two-dimensional grid of metagenes with unsupervised clustering into modules of co-regulated genes. These modules reflect the intrinsic co-variance landscape of the system in gene space. SOM portrayal possesses a series of advantages compared with alternative methods such as NMF or WCGNA (Wirth et al., 2011). Particularly, SOM-portrayal offers a comprehensive downstream analysis pipeline including different options for class discovery in sample and gene space, differential gene expression analysis, function and knowledge mining using gene set analysis with an implemented repository of more than five thousand gene signatures (Löffler-Wirth et al., 2015). SOM portrayal considers the multidimensional nature of gene regulation and pursues a modular view on co-expression, reduces dimensionality and, most importantly, supports visual perception in terms of individual, case-specific expression portraits. The pipeline has been applied to a series of data types and issues, e.g., in the context of molecular oncology (Binder et al., 2022; Loeffler-Wirth et al., 2022; Ashekyan et al., 2023) and health-related population studies (Nikoghosyan et al., 2019; Schmidt et al., 2020a), which all have proven the analytic strength of the methods in complex, multi-dimensional omics data. In the context of vine genomics, oposSOM has been applied so-far as “SOMmelier” to microarray SNP data to discover the dissemination history of Vitis vinifera as seen by vine genomes (Nikoghosyan et al., 2020). The main obstacle for applying the program to transcriptomic data of vine is the lack of gene annotations of their functional context. We here provide the first adaptation of oposSOM to vine transcriptomic data. We employed the SOM algorithm to uncover associations among the cold acclimation mechanisms that have not been characterized before.

In the context of grapevine species grown under different temperature conditions, SOM could help to identify differentially expressed genes of the thiamine biosynthetic pathway in response to temperature changes, providing valuable insights into molecular-level adaptations. Our analysis of transcriptomic mechanisms of temperature adaptation is in line with major questions that must be answered in the context of modern breeding practices nicknamed as Breeding 4.0 (Wallace et al., 2018), namely how do we adapt crops to better fit agricultural environments and what is the nature of the diversity upon which breeding can act?

## 2 Materials and methods

### 2.1 Gene expression data

The analysis was performed on normalized gene expression data as Counts Per Million (CPM) published in Londo et al., 2018 (this data can be found here: https://static-content.springer.com/esm/art%3A10.1038%2Fs41438-018-0020-7/MediaObjects/41438_2018_20_MOESM4_ESM.xlsx; downloaded on 6^th^ February 2023). The data was collected from leaves of five *Vitis vinifera L.* cultivars (Cabernet Franc - CabFra, Chardonnay - Chard, Riesling - Riesl, Sangiovese - Sangio, and Tocai Friulano - Tocai), each treated by four different temperature conditions: 21 °C referenced as “warm”, 4°C referenced as “acclim” or chill/cold, 4°C to -3°C referenced as “accfreeze” or freeze-shock, and -3°C referenced as “freeze” (see Materials and Methods of Londo et al., 2018). Each sample was obtained in up to three replicates, thus overall providing 59 RNAseq data sets (one Riesling replicate was not analyzed). Each sample provides transcript abundance values as CPM for 18,367 genes. These represent 52.28% of the 35,134 annotated coding genes of *Vitis vinifera* that were found in Ensembl database (Kinsella et al., 2011; Yates et al., 2022) on 16th of May 2023. Gene IDs were converted from the Grape Gene Reference Catalogue format (V1) to the INTEGRAPE gene annotation format (V3). For the conversion, the publicly available genome annotation file VCost.v3_28_INTEGRAPEv2.gff3 (Canaguier et al., 2017; downloaded on 6th February 2023) was used. After the conversion, cleaning, and filtering for relevant data, the final dataset was shortened by 220 genes to 18,147 genes.

### 2.2 Application of SOM algorithm

SOM, based on the Kohonen map described in the 1980s, is an unsupervised machine learning technique for analyzing covariance patterns in large multidimensional data (Kohonen, 1982). The input data is linked to neurons on a 2-dimensional map via synapses of varying weights connecting it with the neighboring neurons on the map. The algorithm aims to find synaptic values for each neuron and its adjacent neurons that best fit the input data. The synaptic values are adjusted repeatedly until each neuron on the map represents a portion of the input with similar characteristics, and similar neurons cluster together in proximity based on their similarity to the input data. This allows the neurons to establish their position on the map that accurately represents the input data. The interactions between neighboring neurons “self-organize” the map in a way that neighboring neurons show correlated profiles of the input data forming clusters of coregulated genes appearing as “spots” in the two-dimensional images visualizing the map. Hence, once the map is visualized, it reveals the structure of the input data and identifies correlations in gene expression regulation. We utilized the 18,147 gene expression values in log_10-scale after quantile normalization and centralization of 59 samples as input data for SOM training which distributes the gene expression values over 1,600 neurons, also called metagenes. They are arranged in a quadratic lattice of size 40 x 40. SOM-derived expression portraits were obtained by coloring the metagene expression values from blue (low expression) via green (intermediate) to maroon (high) for each sample. Modules of co-expressed genes were extracted as spots from the individual maps. We applied an adjusted version of the program called “oposSOM” with default settings for SOM training and spot detection (Löffler-Wirth et al., 2015). Mean SOM portraits were obtained by averaging metagene expression values over all individual SOM portraits of a certain group such as replicates of each accession or samples referring to one temperature condition.

### 2.3 Functional interpretation of SOM spots and *Vitis vinifera* genes

The spot modules identified from SOM analysis represent clusters of co-expressed genes, which turns SOM portrayal into an unsupervised clustering method because their number as well as the genes per cluster are selected by the segmentation algorithm in dependence on the intrinsic co-variance structure of the expression data. According to the guilt-by-association principle (Quackenbush, 2003), we estimated the functional impact of the spot modules by applying gene set overrepresentation analysis (via oposSOM) and gene ontology (GO) enrichment analysis via the Overrepresentation Test of PANTHER (Thomas et al., 2022, accessed in February 2023; with default settings), followed by the Semantic similarity reduction feature of REVIGO (Supek et al., 2011; accessed in February 2023; with default settings). Gene sets were visualized by CirGO (Kuznetsova et al., 2019). Lists of genes employed in subsequent analyses were retrieved from BRITE (Biomolecular Relationship Information Transfer Encyclopedia) of KEGG (Kanehisa and Goto, 2000; accessed from February to July 2023) and from “All Pathways” dataset of VitisNet (Grimplet et al., 2009; accessed in February 2023). Genes referred to as epigenetic factors were extracted from BRITE 03036 “Chromosome and Associated Proteins” (Eukaryotic type).

## 3 Results

### 3.1 Gene expression changes as a function of temperature conditions

**Figure 1:**
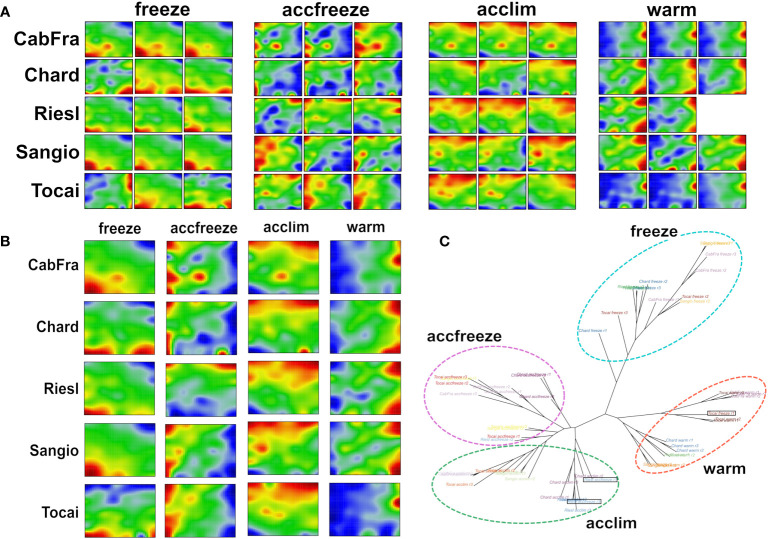
SOM portrayal and sample similarity analysis reveal clearly distinct patterns among the different temperatures. **(A)** Individual SOM portraits of all replicates (red color = overexpressed gene, blue color = underexpressed gene). **(B)** Replicate-averaged SOM portraits. **(C)** The neighbor joining tree splits into four major branches referring to the four temperature-based clusters. Out-grouped samples are in black rectangles.

The SOM algorithm generated sample-specific “portraits” of the normalized gene expression data obtained in triplicate from the leaves of five grapevine cultivars each treated by four different temperature conditions (**Figure 1A**). The different cultivars and the replicates revealed four distinguishable patterns related to the temperature conditions applied (see replicate-averaged portraits in **Figure 1B** and the tree in **Figure 1C**). However, three distinct samples stand out from the others based on their temperature conditions, namely Tocai in the freeze condition (replicate 1), and Riesling in the accfreeze condition (replicates 1 and 3). Surprisingly, the latter two samples do not cluster together with the other samples that share the same temperature condition, but instead, they cluster with the group of samples from the acclim condition. The Tocai “outlier freeze” sample clusters with samples from the warm conditions. Notably, the SOM portraits not only visualize the gene expression changes between the different stress conditions in terms of characteristic spot patterns but also characterize the nature of the outlier samples: The portraits of the two Riesling accfreeze replicates 1 and 2 resemble replicate 3 but in addition show a spot characteristic for the acclim portraits. The portrait of the Tocai freeze replicate 1 clearly agrees with the three warm condition replicates. This way SOM portrayal identifies outlier samples and provides an idea about the affected gene expression programs suggesting transition states, e.g., due to incomplete equilibration (Riesling) or other unknown factors (Tokai).

**Figure 2:**
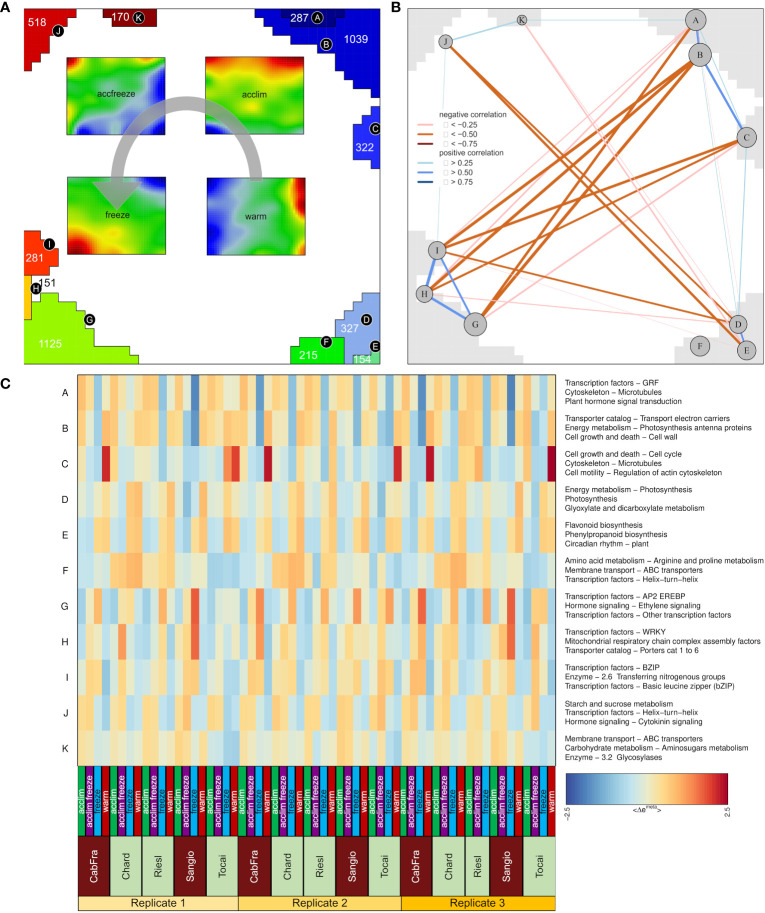
Description of metagenes. **(A)** Group overexpression spots (A-K) and four portraits merged by temperature; Gray arrow indicates spot assignment to the temperature portraits; Numbers inside spots (for H = 151) represent numbers of genes. **(B)** Spot correlation identified by weighted topological overlap algorithm. **(C)** Group overexpression spots patterns among samples with the top three gene sets characteristic for each spot.

Next, we calculated mean portraits for each of the four temperature conditions averaged over the respective cultivars and replicated samples to extract the respective condition-specific expression patterns (**Figures 1B**; **2A**). The red spot-like areas represent modules of co-expressed genes activated at the respective condition. One sees that overexpression modules rotate in counterclockwise direction from the right edge of the map (up at warm condition) via the upper edge (up at acclim and accfreeze) towards the left lower corner (up in freeze). For further downstream analysis we make use of the spot selection function implemented in the oposSOM package which has identified eleven distinct spot modules labeled A-K as indicated in **Figure 2A** and in detail present in **Supplementary Table S1**. The organization of spots within the portrait’s is governed by the temperature conditions and can be characterized by their mutual correlation network: For example, spot modules A-C, activated at warm and acclim conditions, mutually correlate but anti-correlate with the expression of spot-modules I, G and H up at freeze conditions (**Figure 2B**). The spot-module expression across all cultivars, conditions and replicates is summarized in **Figure 2C**. Each spot module contains between 151 (spot H) and 1125 (spot G) single genes. For functional characterization we applied gene set analysis provided by more than 400 sets from VitisNet and BRITE (**Figure 2C**).

The spots A to F that were assigned with warm and acclim conditions contain genes that are intrinsically associated with a multitude of normal physiological processes, for instance photosynthesis, flavonoid biosynthesis, linoleic acid biosynthesis, phenylpropanoid biosynthesis, amino acid metabolism, circadian rhythm regulation, DNA transcription, replication, repair mechanisms, and a variety of enzymatic reactions. In spots G through K, assigned with accfreeze and freeze conditions, there is a prevalence of genes that are primarily involved in processes indicating plant response to stress, such as pathogen response mechanism, carbohydrate metabolism, nitrogen metabolism, and hormonal signaling pathways.

Two spots, B and G, contain the most genes per spot. Given that spot B contains the samples grown in the warm and acclim conditions and the samples occupying spot G represents accfreeze and freeze conditions, a comparative analysis of gene functions within these two spots could yield significant insights. Spot B is characterized by processes such as electron transport, cell growth, cell wall formation, porphyrin metabolism, thylakoid pathway regulation, and auxin signaling. Additionally, genes involved in chlorophyll binding and the establishment of the cytoskeleton are also present. Many of the processes in spot B are tightly bound to photosynthesis and normal plant growth. Conversely, spot G, which is specific to samples grown under freezing conditions, activates genes coding for AP2, WRKY, NAC, and MYB transcription factors. Other overexpressed genes of the spot G relate to the ethylene and auxin signaling pathways, energy metabolism, and plant-pathogen interaction. This suggests that the plant employs an enhanced resistance system to mitigate the damaging effects of freezing stress.

It is important to highlight that the majority of processes in warm and acclim conditions are fundamentally linked to the photosynthetic processes and in general to the standard developmental trajectory of plants. On the other hand, most of the processes in the samples subjected to freezing temperatures are directly or indirectly related to the plant’s stress response. This is particularly evident in the activation of pathogen-responsive genes which play a crucial role in the plant’s defense mechanism against external stressors.

SOM discerned a distinct dichotomy in gene expression patterns across varying temperature conditions in the studied samples. Specifically, spots A-F and G-K, representative of warm and freeze conditions respectively, exhibited a high specificity of processes integral to either photosynthesis and plant growth (warm), and stress response mechanisms (freeze), thereby providing a comprehensive characterization of plant adaptation strategies to temperature stress.

### 3.2 Plant response to cold stress activates thiamine biosynthesis

**Figure 3:**
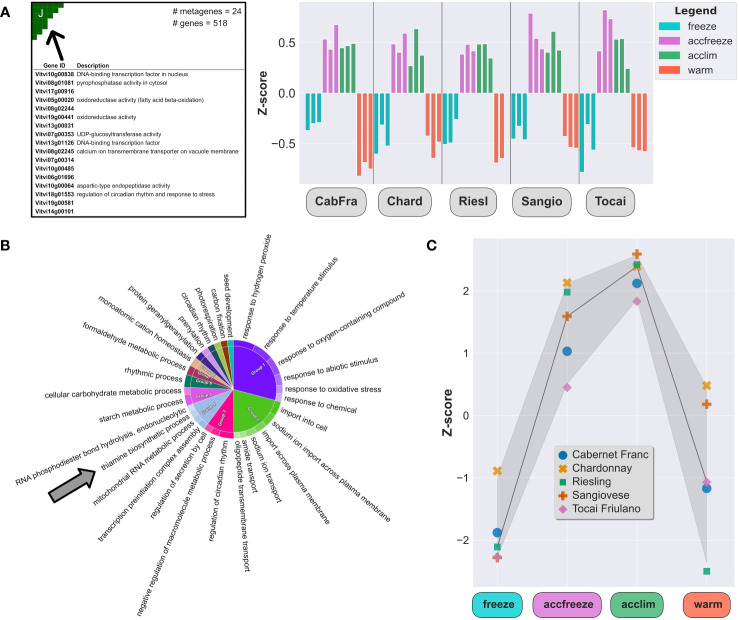
Chill shock-stressed plants involve thiamine metabolism in their response to temperature change. **(A)** Top 20 overexpressed genes description (left) and Z-scores (right) of all overexpressed genes in the spot J (indicated by arrow). **(B)** The most enriched GO terms in a set of 518 genes from the spot J; arrow indicates enrichment by the thiamine biosynthesis GO. **(C)** Z-scores of the thiamine metabolism gene set (22 genes) in each sample.

In the samples derived from acclim and accfreeze plants, a significant portion of overexpressed genes was found in spot J (**Figure 3A**). To gain a deeper understanding of the biological functions of these genes, we conducted gene ontology enrichment analysis (**Figure 3B**). Plants employ various mechanisms to cope with low temperature stress, and as expected, the most enriched gene ontology terms in the samples were related to biological processes involved in plant response to temperature changes and associated processes, such as response to biotic and abiotic stress, transmembrane transport, circadian rhythm, hormonal regulation, and starch metabolism (Bieniawska et al., 2008; Maruyama et al., 2009; Eremina et al., 2016).

Among the enriched GO terms, we made an unexpected observation of an association with the thiamine biosynthetic process. Considering this finding, we shifted our focus to explore the potential link between temperature control mechanisms and thiamine metabolism and biosynthesis. Furthermore, we noticed that the gene expression of some thiamine biosynthetic enzymes, like THIAMINE THIAZOLE SYNTHASE 1 and 2 (THI1-1 and THI1-2), PHOSPHOMETHYLPYRIMIDINE SYNTHASE (THIC), or probable 1-DEOXY-D-XYLULOSE-5-PHOSPHATE SYNTHASE (DXS), are elevated in the acclim and accfreeze plants when compared to the freeze and warm conditions (**Figure 3C**), which aligns with our hypothesis.

**Figure 4:**
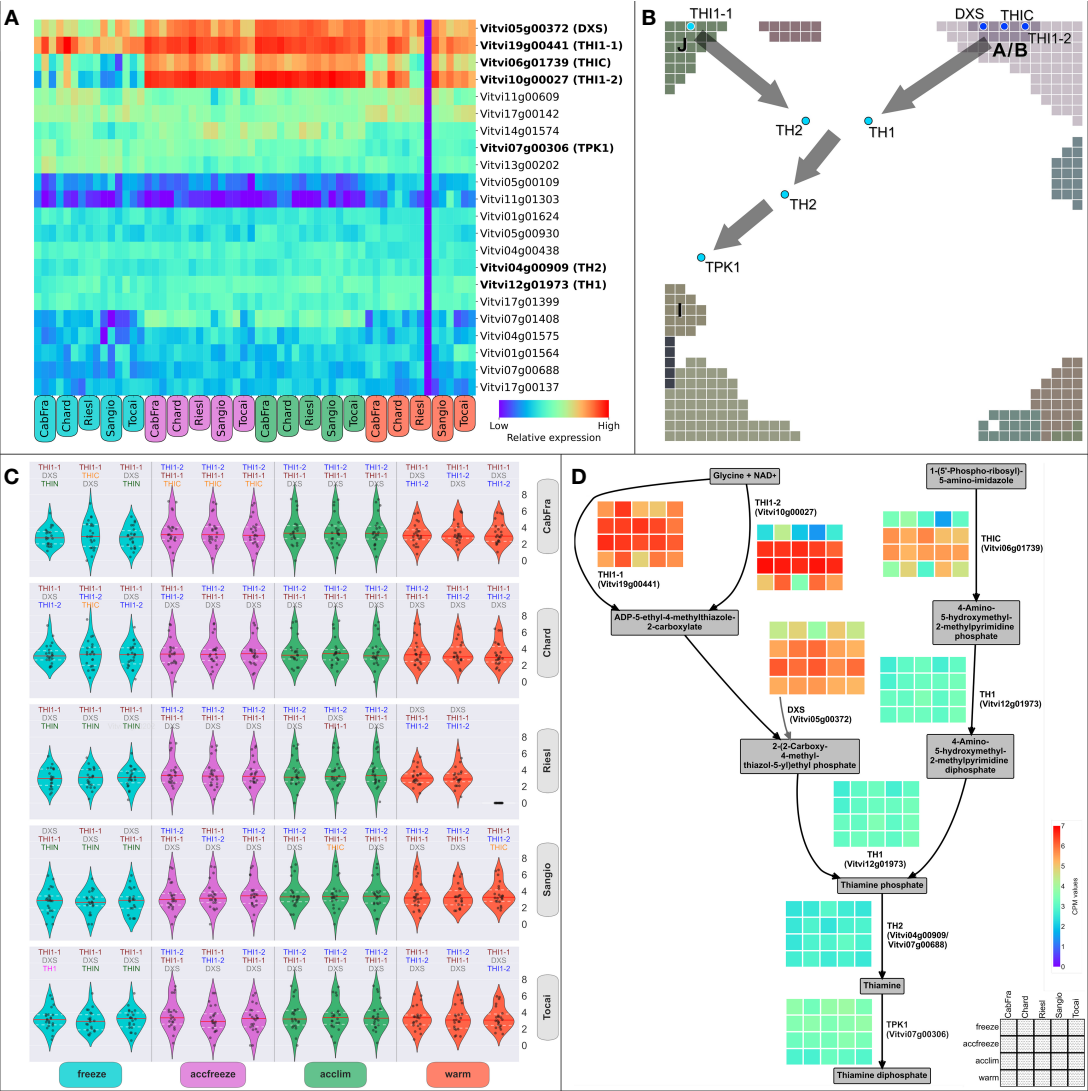
Thiamine metabolic genes and biosynthesis pathway. **(A)** Clustering of genes (by CPM values across samples) involved in thiamine metabolism; highlighted genes code for known enzymes of thiamine biosynthesis. **(B)** Positions of the thiamine biosynthetic genes in the SOM portrait; gray squares = Group Overexpression Spots; gray arrows = direction of the thiamine biosynthetic pathway. **(C)** Distribution of the normalized expression of the thiamine metabolism genes; the three most expressed biosynthetic genes have their names written on top of each violin plot. **(D)** Simplified scheme of the thiamine biosynthesis pathway (including the side branch with DXS); heatmaps display normalized gene expression; legend and color bar are at the bottom right corner; color scaling is normalized to all heatmaps.

The thiamine biosynthetic pathway in plants, reviewed in Guan et al., 2014, begins with the enzyme THIC, followed by CYSTEINE-DEPENDENT ADENOSINE DIPHOSPHATE THIAZOLE SYNTHASE (THI1), which is encoded by two genes, *THI1-1* (*Vitvi19g00441*) and *THI1-2* (*Vitvi10g00027*). The product of THI1 activity is synthesized after certain precursors from glycolysis are catalyzed by DXS. The pathway continues with the enzyme THIAMINE BIOSYNTHETIC BIFUNCTIONAL ENZYME (TH1), which carries out multiple functions critical to thiamine biosynthesis. The synthesis of thiamine is completed with the involvement of the enzyme THIAMINE PHOSPHATE PHOSPHATASE/AMINO-HMP AMINOHYDROLASE (TH2), encoded by two distinct genes. Finally, the enzyme THIAMINE PYROPHOSPHOKINASE (TPK1; also referred as THIN) converts thiamine into its active form, thiamine diphosphate. To gain insights into the regulatory mechanisms governing the biosynthesis of thiamine, our investigation centered on an in-depth analysis of the genes orchestrating the catalysis of pivotal reactions within the thiamine biosynthetic pathway (**Figure 4A**). Genes *THI1-1*, *THI1-2*, *THIC* (*Vitvi06g01739*), and *DXS* (*Vitvi05g00372*) are crucial as they encode enzymes that catalyze the initial steps of the pathway (**Figure 4D**). Therefore, any changes in their expression levels can significantly impact the overall production of thiamine. The gene expression data revealed that the activity of these four genes is elevated in the accfreeze and acclim samples (**Figure 4A**).

Next, we endeavored to shed light on the intricate interplay of these enzyme-coding genes, which are fundamental for shaping the production of thiamine. Utilizing SOM modularization of the expression landscape, we have unraveled a captivating phenomenon in the realm of thiamine biosynthesis, where the selection of specific enzymes crucial for this process is intricately linked to prevailing temperature conditions (**Figure 4B**). These highly expressed genes are in spots A/B and J, indicating two metabolic origins (**Figures 4B, D**). Notably, we observed a higher level of gene expression of *THI1-1* (spot J) during the freeze and warm conditions, while the *THI1-2* (spot A/B) gene takes precedence in acclim and accfreeze conditions (**Figures 4A, C**). The genes *TH1* (*Vitvi12g01973*), *TH2* (*Vitvi04g00909*/*Vitvi07g00688*), and *TPK1* (*Vitvi07g00306*), downstream of the thiamine metabolic pathway, continue the path in the portrait either from the spot A/B (“warm origin”) or from the spot J (“cold origin”), converging in a spotless midpoint between the acclim and accfreeze areas of the portrait, and ending nearby the spot I area (**Figure 4B**). Interestingly, such topology as described in **Figure 4B** also resembles the hierarchical structure of the thiamine pathway (**Figure 4D**), with the reactions catalyzed either by THIC, THI1-2, and DXS (“warm origin”-based) or THI1-1 (“cold origin”-based), both converging at the step of synthesis of thiamine phosphate.

Hereby, SOM reveals that acclimated and freeze-acclimated plants overexpress genes related to temperature changes, including an association with thiamine biosynthesis. The expression of key thiamine biosynthetic enzymes was found to be elevated in the accfreeze samples, indicating a link between thiamine and temperature control. Additionally, SOM provided an exclusive insight into the “temperature cascade”-like gene topology derived purely from the gene expression data.

### 3.3 Temperature shapes activity of genes coding epigenetic factors in *Vitis vinifera*

Epigenetic mechanisms, encompassing DNA and/or histone modifications and the modulation of chromatin accessibility, have been compellingly demonstrated to regulate the expression of stress-responsive genes in the face of abiotic challenges, including the response to low temperatures (Xie et al., 2017).

**Figure 5:**
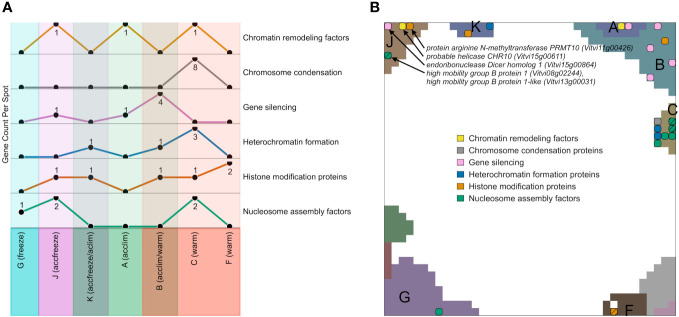
Expression topology of genes related to epigenetics. **(A)** The abundance of genes encoding epigenetic factors from different spots related to different temperatures. Spots that do not contain any overexpressed epigenetic genes are not shown. **(B)** Distribution of genes encoding epigenetic factors involved in chromatin remodeling, chromosome condensation, gene silencing, heterochromatin formation, histone modifications, or nucleosome assembly across the spots in the SOM hatch indicates overlapping epigenetic processes at the same position. A full list of epigenetic factors across the spots is provided in **Supplementary Table S2**.

We filtered genes encoding epigenetic factors in the gene lists of the different expression spots, plotted their number across the spots and mapped them into the SOM (**Figures 5A, B**). Among the spots containing genes upregulated in warm conditions, most factors related to chromatin condensation, gene silencing, heterochromatin formation, and nucleosome assembly accumulate in spot C and B, while the number of genes for histone modification factors is the highest in the spot F. Spots that are activated in accfreeze, and acclim samples contain less epigenetic factors than the warm samples, while epigenetic factors are absent in the spot modules activated under freeze conditions. In other words, this overall decaying amount reflects a decaying impact of epigenetic regulation under stress and especially at freezing conditions.

Epigenetic regulation modes play a certain role also at accfreeze conditions (spot J and partly K). Some genes coding for gene silencing, histone modification proteins, chromatin remodeling factors, and nucleosome assembly factors are overexpressed in at least one of those spots. On the other hand, genes coding for histone modification proteins are spread among spots F, B, C, K, and J, showing that such genes must be highly expressed not only in warm conditions, but also in the cold (acclim) and freeze shock (accfreeze).

Hence, the data confirms elevated activity of various genes involved in epigenetic regulation across temperature conditions which highlights the remarkable adaptability of plants to temperature changes, modulated by epigenetic mechanisms. The information about epigenetic genes in spot J suggests their potential roles in plant response to low temperatures.

## 4 Discussion

In this study, we reanalyzed the data from Londo et al., 2018 by SOM (Kohonen, 1982; Löffler-Wirth et al., 2015), which is a machine learning technique that allows the visualization and clustering of high-dimensional data. Our objective was to identify the clusters of genes that were associated with specific temperature conditions (namely warm, acclim, accfreeze, and freeze) and to investigate the gene ontology terms associated with the overexpression of genes in the samples grown under such conditions with the special focus on stressed accfreeze.

Analysis of gene expression data, typically containing information about thousands of genes expressed under varying conditions, poses significant challenges due to their high-dimensional nature. Traditional methods often struggle with the visualization and interpretation of such complex data. Moreover, identifying clusters of genes with similar expression patterns across different temperature conditions can be a daunting task. The SOM portrayal method, an artificial neural network algorithm, addresses these issues effectively. It offers dimensionality reduction, enabling a low-dimensional representation of the high-dimensional gene expression data. SOM also provides a topological visualization of the data, preserving the original data’s metric relationships, which helps to identify complex patterns in gene expression data. As a non-supervised clustering method, SOM identifies clusters of genes with similar expression patterns. These clusters can be further analyzed to comprehend their functional roles.

Here, for the first time in the analysis of plant gene expression data, we applied SOM portrayal approach. Our analysis revealed specific responses of the gene expression patterns in *Vitis vinifera* to temperature, as demonstrated by the clustering of genes into four major groups, each associated with one of the specific temperature conditions. The SOM algorithm allowed us to visualize the gene expression patterns in a two-dimensional space, where genes with similar expression patterns were grouped together. This “portrayal” of individual expression landscapes identified some replicates, namely Tocai freeze replicate 1 and Riesling accfreeze replicates 1 and 3, showed different SOM portraits than expected according to the other replicates and samples by unknown reasons. Our SOM portrayal approach, on the other hand, confirms previous results of Londo and colleagues, namely that the genes related to hormonal signaling, secondary metabolism, sugar and starch synthesis, and transcription factors from various families (*NAC*, *WRKY*, and *AP2*) were upregulated in the accfreeze samples, compared to the acclim conditions.

Interestingly, we identified another group of genes that were overexpressed in the accfreeze condition. These genes are related to thiamine biosynthesis. The upregulation of thiamine biosynthesis might serve the grapevine plants not only as pathogen protection but also as a crucial mechanism that aids the plants in coping with cold stress, allowing them to better withstand such conditions. This novel information may drive future efforts to improve vine acclimation to cold stress and significantly enhance the viticulture with novel strategies of vine growth. For example, the identification of genes, like *THIC*, *THI1-1*, or *THI1-2*, related to thiamine biosynthesis that are upregulated in the accfreeze condition may provide targets for the development of new treatments or interventions aimed at enhancing plant growth and protection.

Arabidopsis controls the levels of *THIC* transcripts through various regulatory mechanisms. These include responses to light (Raschke et al., 2007), interactions with the circadian clock (Bocobza et al., 2013), and the presence of a riboswitch located in the 3′-UTR of *THIC* mRNA (Sudarsan et al., 2003; Bocobza et al., 2007; Wachter et al., 2007; Bocobza et al., 2013). All these factors are crucial in regulating the biosynthesis of thiamine. According to the currently accepted model, the riboswitch undergoes alternative splicing in the 3′-UTR region, leading to the formation of transcripts with varying 3′-UTR lengths. This splicing event has a direct impact on the stability of the *THIC* mRNA (Bocobza et al., 2007; Wachter et al., 2007). In cassava, the *THIC* and *THI1* genes are expressed at very low levels in storage roots (Mangel et al., 2017). In leaves, vitamin B1 content is negatively correlated with *THIC* and *THI1* expression levels, suggesting post-transcriptional regulation of THIC accumulation by a riboswitch present in the 3′-UTR of the *THIC* mRNA and regulation of *THI1* by promoter activity or alternative post-transcriptional mechanisms. The potential involvement of these mechanisms in determining the dynamic changes in the overexpression of certain thiamine biosynthetic genes and preferential activation of either *THI1-1* or *THI1-2* in *Vitis vinifera* remain to be determined.

Londo and colleagues also showed that the ability of grapevine plants to cope with freezing temperatures (-3°C) when pretreated by 4°C for 48 hours is limited. Physiological differences between non-pretreated (freeze) and freeze-pretreated (accfreeze) plants were vast, the latter condition produced more damaged plants (Londo et al., 2018). We also sought to identify genes coding for epigenetic factors in cold/freezing stress conditions, providing valuable information about the activation of epigenetic mechanisms in different temperatures. The numbers of genes coding for epigenetic factors varied in each spot. This finding suggests different activation of epigenetic genes in changing temperatures. The warm samples, assigned by SOM to the spot C, contain many genes involved in accumulation of nucleosome assembly factors, chromosome condensation, and heterochromatin formation; such processes are important for nucleosome formation, chromatin compaction, and genome stability. This might suggest that such epigenetic processes favor the standard temperature conditions. Nevertheless, this would imply that either the downregulation of the respective genes in cold/freezing stress is required or the regulation of their expression might be sensitive to the stress originating from freezing conditions. In summary, the overexpression of such genes is either not demanded under stress or cannot be upregulated due to freezing conditions.

On the other hand, there is a decent number of the overexpressed genes with epigenetic function in the freeze-shocked (accfreeze) samples. The PROTEIN ARGININE N-METHYLTRANSFERASE 10 (PRMT10) protein, catalyzing the asymmetric di-methylation of arginine 3 on histone 4 which is associated with a more accessible chromatin structure and thus higher levels of transcription, is known to be important for maintaining pleiotropic development and adaptation to abiotic stresses in plants (Niu et al., 2007). By indirectly regulating *FLC* (*FLOWERING LOCUS C*), PRMT10 exerts its influence on floral transition. FLC is a MADS-box transcription factor gene that plays a role in the regulation of cold stress in plants. Vernalization is an example of somatic stress memory where changes in the chromatin structure of the *FLC* gene maintain cold stress memory during mitosis. *FLC* expression suppresses flowering at high levels during winter, and during vernalization, B3 transcription factors, cold memory cis-acting element and POLYCOMB REPRESSIVE COMPLEX 1 and 2 silence *FLC* activation (Kim, 2023). Unfortunately, we did not find any evidence that PRMT10 or any other histone methyltransferase would affect directly or indirectly the production of any of the thiamine biosynthetic enzymes. RNA helicases and their role in cold stress response in plants are known. For example, REGULATOR OF CBF GENE EXPRESSION 1 is a cold-inducible RNA helicase with crucial role in cold tolerance in Arabidopsis (Guan et al., 2013). However, the role of the RNA helicase CHROMATIN REMODELING 10 (CHR10) in plant response to cold stress has not been identified. In Arabidopsis, the transcript level of *HIGH MOBILITY GROUP B PROTEIN 1* remains unchanged when exposed to cold temperatures (Kwak et al., 2007). The ENDORIBONUCLEASE DICER HOMOLOG 1 enzyme, catalyzing RNA cleavage, influences the cold response indirectly via its role in regulating miRNA biogenesis (Huo et al., 2022). However, there is no information about its direct involvement in response to cold stress. Additional experimental investigation at molecular level might elucidate the significance of epigenetic mechanisms in regulation of cold/freeze temperature stress response in *Vitis vinifera*. Such research would contribute to a deeper understanding of the molecular processes involved in the plant’s adaptive response to environmental challenges.

Overall, the use of machine learning-based SOM portrayal pipeline (Binder & Wirth, 2014) in vine omics data analyses represents a promising advancement in the field of plant molecular biology and genetics. It allows for the visualization and clustering of high-dimensional omics data and their “portrayal” in gene space, making it possible to identify complex patterns reflecting pathway activation and transcriptional trajectories describing temperature response in a topology aware manner (see **Figures 1**-**3**, and Schmidt et al., 2020b for the concept). This can lead to the detection of novel associations and insights previously hidden in the data’s complexity due to the high resolution and sensitivity of the method to detect modules of co-regulated genes (Loeffler-Wirth et al., 2019). Notably, SOM portrayal provides an expression landscape of all genes under study which improves functional interpretation because this approach also considers the effect of weak expression changes (**Figure 4**). Importantly, SOM-portrayal visualizes the dimension-reduced gene state space while other methods such as principal component analysis (PCA), t-Distributed Stochastic Neighbor Embedding (t-SNE), Uniform Manifold Approximation and Projection (UMAP) and related methods typically apply to sample state space (**Supplementary Image S3**; Gardner et al., 2020; Gorgoglione et al., 2021). SOM portrayal therefore provides an orthogonal, gene-centric view on high dimensional omics data complementing sample-centered dimension reduction methods (Hopp et al., 2013).

The identification of genes related to thiamine biosynthesis that are upregulated in cold stress conditions could provide targets for the development of new treatments or interventions aimed at enhancing plant growth and protection. This could lead to improved plant acclimation to cold stress, significantly enhancing viticulture with novel strategies of vine growth. Moreover, understanding the role of epigenetic mechanisms in regulating the expression of stress-responsive genes in response to low temperatures could provide valuable insights into the plant’s adaptive response to environmental challenges. This knowledge could be harnessed to develop new strategies for improving the resilience of grapevines to temperature stress, thereby contributing to sustainable viticulture practices in the face of climate change.

## Data availability statement

Publicly available datasets were analyzed in this study. This data can be found here: https://static-content.springer.com/esm/art%3A10.1038%2Fs41438-018-0020-7/MediaObjects/41438_2018_20_MOESM4_ESM.xlsx.

## Author contributions

TK: Conceptualization, Formal analysis, Methodology, Software, Writing – original draft, Writing – review & editing. MN: Writing – review & editing. HB: Conceptualization, Funding acquisition, Methodology, Supervision, Writing – original draft, Writing – review & editing.
