# Supplementary material for: Machine learning extracts marks of thiamine’s role in cold acclimation in the transcriptome of Vitis vinifera
Source: Front Plant Sci. 2023 Dec 6;14:1303542. doi: 10.3389/fpls.2023.1303542 (PMC10731266; doi:10.3389/fpls.2023.1303542)

Supplementary Material

**Supplementary Table S1.** Description of the spots A-K with the top 10 overrepresented gene sets and the 10 most strongly correlated genes with each spot.

| **Spot (condition)** | **Meta-** **gene** | **Overrepresented gene sets** | **Gene rank** | **Gene ID** | **Mean expr.** | **SD** | **Correlation** | **p-value** |
| --- | --- | --- | --- | --- | --- | --- | --- | --- |
| A (acclim) | 34 x 40 | Transcription factors GRF | 1 | *Vitvi02g00532* | 3.15 | 1.31 | 0.68 | 1.30E-09 |
| A (acclim) | 35 x 40 | Microtubules | 2 | *Vitvi04g01873* | 4.53 | 1.48 | 0.86 | 0.00E+00 |
| A (acclim) | 33 x 40 | Plant hormone signal transduction | 3 | *Vitvi15g01388* | 3.06 | 1.51 | 0.63 | 4.84E-08 |
| A (acclim) | 33 x 40 | Transcription factors ARF | 4 | *Vitvi16g01176* | 2.38 | 1.39 | 0.72 | 5.69E-11 |
| A (acclim) | 35 x 39 | Regulation of actin cytoskeleton | 5 | *Vitvi18g02045* | 1.69 | 1.27 | 0.51 | 2.18E-05 |
| A (acclim) | 34 x 40 | Phagosome | 6 | *Vitvi14g03036* | 3.85 | 1.18 | 0.74 | 9.94E-12 |
| A (acclim) | 36 x 40 | Phagosome | 7 | *Vitvi09g00593* | 2.41 | 1.30 | 0.83 | 3.33E-16 |
| A (acclim) | 30 x 40 | Thiamine metabolism | 8 | *Vitvi18g03009* | 1.61 | 1.24 | 0.54 | 4.83E-06 |
| A (acclim) | 35 x 40 | Exosome | 9 | *Vitvi10g00027* | 5.79 | 1.96 | 0.88 | 0.00E+00 |
| A (acclim) | 33 x 40 | Peptidases and inhibitors: pepsin family | 10 | *Vitvi10g01138* | 3.04 | 1.46 | 0.77 | 4.33E-13 |
| B (acclim) | 40 x 39 | Transport electron carriers | 1 | *Vitvi14g01929* | 3.29 | 1.87 | 0.83 | 2.22E-16 |
| B (acclim) | 40 x 36 | Photosynthesis antenna proteins | 2 | *Vitvi09g01282* | 2.11 | 1.83 | 0.64 | 1.93E-08 |
| B (acclim) | 40 x 40 | Cell wall | 3 | *Vitvi13g00172* | 3.43 | 2.11 | 0.88 | 0.00E+00 |
| B (acclim) | 40 x 34 | Photosynthesis antenna proteins | 4 | *Vitvi19g02024* | 1.07 | 1.21 | 0.55 | 3.51E-06 |
| B (acclim) | 40 x 39 | Energy metabolism | 5 | *Vitvi17g01251* | 2.80 | 1.84 | 0.84 | 1.11E-16 |
| B (acclim) | 40 x 40 | Photosynthesis | 6 | *Vitvi13g01337* | 2.84 | 1.75 | 0.91 | 0.00E+00 |
| B (acclim) | 40 x 40 | Photosystem I (P700 chlorophyll a) | 7 | *Vitvi06g01346* | 4.31 | 2.33 | 0.96 | 0.00E+00 |
| B (acclim) | 40 x 39 | Porphyrin metabolism | 8 | *Vitvi19g00680* | 1.83 | 1.60 | 0.82 | 1.55E-15 |
| B (acclim) | 40 x 40 | Regulation of actin cytoskeleton | 9 | *Vitvi10g01636* | 3.07 | 2.19 | 0.87 | 0.00E+00 |
| B (acclim) | 40 x 40 | Thylakoid targeting pathway | 10 | *Vitvi17g00601* | 3.78 | 2.16 | 0.90 | 0.00E+00 |
| C (warm) | 40 x 24 | Cell cycle | 1 | *Vitvi03g00752* | 3.95 | 1.90 | 0.51 | 2.21E-05 |
| C (warm) | 40 x 26 | Microtubules | 2 | *Vitvi18g02927* | 0.97 | 1.77 | 0.92 | 0.00E+00 |
| C (warm) | 40 x 26 | Regulation of actin cytoskeleton | 3 | *Vitvi11g01227* | 0.89 | 1.61 | 0.92 | 0.00E+00 |
| C (warm) | 40 x 26 | DNA Replication Initiation Factors | 4 | *Vitvi11g01222* | 1.29 | 1.64 | 0.89 | 0.00E+00 |
| C (warm) | 40 x 26 | DNA replication | 5 | *Vitvi07g02007* | 0.91 | 1.54 | 0.91 | 0.00E+00 |
| C (warm) | 40 x 26 | Replication and repair | 6 | *Vitvi01g00742* | 1.35 | 1.72 | 0.88 | 0.00E+00 |
| C (warm) | 40 x 25 | DNA Replication Termination Factors | 7 | *Vitvi07g02362* | 2.13 | 1.74 | 0.59 | 3.78E-07 |
| C (warm) | 40 x 26 | Homologous recombination | 8 | *Vitvi18g01488* | 0.84 | 1.41 | 0.89 | 0.00E+00 |
| C (warm) | 40 x 26 | Base excision repair | 9 | *Vitvi11g01224* | 0.84 | 1.35 | 0.89 | 0.00E+00 |
| C (warm) | 40 x 29 | Mismatch repair | 10 | *Vitvi04g00312* | 1.10 | 1.54 | 0.63 | 3.71E-08 |
| D (warm) | 40 x 3 | Energy metabolism | 1 | *Vitvi03g00325* | 1.53 | 1.46 | 0.76 | 2.29E-12 |
| D (warm) | 40 x 3 | Photosynthesis | 2 | *Vitvi13g00369* | 1.44 | 1.27 | 0.67 | 2.33E-09 |
| D (warm) | 40 x 5 | Glyoxylate and dicarboxylate metabolism | 3 | *Vitvi13g02005* | 3.97 | 1.54 | 0.65 | 1.66E-08 |
| D (warm) | 37 x 3 | Enzymes acting on a sulfur | 4 | *Vitvi01g01981* | 2.62 | 1.68 | 0.68 | 1.24E-09 |
| D (warm) | 36 x 5 | Nitrogen metabolism | 5 | *Vitvi10g00020* | 2.81 | 1.06 | 0.50 | 2.66E-05 |
| D (warm) | 38 x 3 | Nitrogen metabolism | 6 | *Vitvi16g00731* | 0.81 | 1.10 | 0.80 | 1.95E-14 |
| D (warm) | 40 x 4 | Transporters 30 to 64 | 7 | *Vitvi12g02451* | 0.76 | 0.85 | 0.78 | 2.05E-13 |
| D (warm) | 40 x 7 | Flavonoid biosynthesis | 8 | *Vitvi05g01116* | 3.09 | 0.99 | 0.45 | 1.75E-04 |
| D (warm) | 40 x 4 | Linoleic acid metabolism | 9 | *Vitvi14g01336* | 1.75 | 0.97 | 0.72 | 9.28E-11 |
| D (warm) | 40 x 5 | Photosynthetic electron transport | 10 | *Vitvi19g00302* | 1.94 | 1.31 | 0.80 | 1.71E-14 |
| E (warm) | 40 x 1 | Flavonoid biosynthesis | 1 | *Vitvi00g00346* | 1.38 | 1.58 | 0.85 | 0.00E+00 |
| E (warm) | 40 x 1 | Phenylpropanoid biosynthesis | 2 | *Vitvi07g02904* | 1.38 | 1.58 | 0.85 | 0.00E+00 |
| E (warm) | 39 x 1 | Circadian rhythm | 3 | *Vitvi02g01118* | 0.69 | 0.81 | 0.69 | 8.78E-10 |
| E (warm) | 38 x 1 | Transcription factors (Helix−turn−helix) | 4 | *Vitvi13g00870* | 2.54 | 1.25 | 0.33 | 4.91E-03 |
| E (warm) | 40 x 2 | Endoplasmic reticulum and cytosol | 5 | *Vitvi11g01421* | 2.29 | 1.27 | 0.68 | 2.27E-09 |
| E (warm) | 40 x 1 | Methane metabolism | 6 | *Vitvi02g00110* | 1.83 | 1.05 | 0.73 | 3.20E-11 |
| E (warm) | 40 x 2 | Transcription factors MYB | 7 | *Vitvi05g02017* | 1.98 | 1.37 | 0.73 | 2.69E-11 |
| E (warm) | 40 x 1 | Chaperone HSP70 / DNAK | 8 | *Vitvi15g01070* | 1.61 | 1.01 | 0.77 | 7.26E-13 |
| E (warm) | 40 x 2 | Proteasome assembling factors | 9 | *Vitvi01g01980* | 2.67 | 1.28 | 0.80 | 2.44E-14 |
| E (warm) | 40 x 1 | Transporters 66 to 94 | 10 | *Vitvi11g01303* | 0.77 | 0.79 | 0.71 | 2.10E-10 |
| F (warm) | 36 x 1 | Arginine and proline metabolism | 1 | *Vitvi12g02565* | 0.74 | 1.23 | 0.36 | 2.51E-03 |
| F (warm) | 31 x 1 | ABC transporters | 2 | *Vitvi09g02008* | 0.81 | 1.12 | 0.45 | 1.64E-04 |
| F (warm) | 33 x 1 | Transcription factors (Helix−turn−helix) | 3 | *Vitvi04g01863* | 1.08 | 1.70 | 0.57 | 9.84E-07 |
| F (warm) | 33 x 1 | Tyrosine metabolism | 4 | *Vitvi11g01446* | 1.23 | 1.61 | 0.67 | 2.54E-09 |
| F (warm) | 31 x 1 | Ribosome biogenesis | 5 | *Vitvi11g01457* | 2.45 | 1.44 | 0.50 | 2.61E-05 |
| F (warm) | 33 x 1 | Transcription factors HSF | 6 | *Vitvi10g01863* | 0.89 | 1.35 | 0.66 | 5.05E-09 |
| F (warm) | 33 x 1 | Photosynthesis | 7 | *Vitvi10g01433* | 2.28 | 1.61 | 0.66 | 6.15E-09 |
| F (warm) | 34 x 1 | Ubiquinone biosynthesis | 8 | *Vitvi17g00339* | 1.56 | 1.35 | 0.62 | 6.62E-08 |
| F (warm) | 33 x 1 | Replication and mismatch repair | 9 | *Vitvi08g01434* | 0.97 | 0.94 | 0.65 | 9.56E-09 |
| F (warm) | 34 x 1 | Homologous recombination | 10 | *Vitvi10g00378* | 1.41 | 1.57 | 0.55 | 3.08E-06 |
| G (freeze) | 5 x 1 | Transcription factors AP2 EREBP | 1 | *Vitvi06g01713* | 1.64 | 1.52 | 0.77 | 5.24E-13 |
| G (freeze) | 4 x 1 | Ethylene signaling | 2 | *Vitvi05g00204* | 1.89 | 2.30 | 0.86 | 0.00E+00 |
| G (freeze) | 5 x 1 | Other transcription factors | 3 | *Vitvi15g00835* | 1.40 | 1.80 | 0.80 | 1.09E-14 |
| G (freeze) | 4 x 3 | Transcription factors WRKY | 4 | *Vitvi09g01554* | 1.36 | 1.58 | 0.78 | 1.93E-13 |
| G (freeze) | 3 x 1 | Transcription factors NAC | 5 | *Vitvi05g00170* | 2.57 | 2.42 | 0.91 | 0.00E+00 |
| G (freeze) | 5 x 6 | Galactose metabolism | 6 | *Vitvi18g00353* | 1.87 | 1.50 | 0.43 | 3.14E-04 |
| G (freeze) | 4 x 3 | Transcription factors MYB | 7 | *Vitvi06g01917* | 2.58 | 1.80 | 0.74 | 8.90E-12 |
| G (freeze) | 4 x 4 | Transcription factors (Helix−turn−helix) | 8 | *Vitvi18g03065* | 1.04 | 1.47 | 0.75 | 4.34E-12 |
| G (freeze) | 4 x 2 | Tryptophan metabolism | 9 | *Vitvi06g01280* | 1.77 | 1.69 | 0.83 | 4.44E-16 |
| G (freeze) | 4 x 6 | Auxin biosynthesis | 10 | *Vitvi02g01405* | 1.79 | 1.24 | 0.64 | 2.57E-08 |
| H (freeze) | 1 x 6 | Transcription factors WRKY | 1 | *Vitvi08g00957* | 1.69 | 1.60 | 0.62 | 9.05E-08 |
| H (freeze) | 1 x 8 | Mitochondrial respiratory chain complex | 2 | *Vitvi16g01469* | 1.22 | 1.09 | 0.85 | 0.00E+00 |
| H (freeze) | 1 x 7 | Transporters 1 to 6 | 3 | *Vitvi08g01744* | 1.20 | 1.10 | 0.86 | 0.00E+00 |
| H (freeze) | 1 x 6 | Transcription factors HSF | 4 | *Vitvi16g01213* | 1.30 | 1.10 | 0.81 | 4.66E-15 |
| H (freeze) | 1 x 7 | Receptors | 5 | *Vitvi16g01986* | 1.34 | 1.20 | 0.73 | 3.88E-11 |
| H (freeze) | 1 x 8 | SLC47: Multidrug and Toxin Extrusion | 6 | *Vitvi05g01760* | 1.59 | 1.06 | 0.67 | 4.12E-09 |
| H (freeze) | 1 x 7 | Transporters 66 to 94 | 7 | *Vitvi16g01461* | 1.23 | 1.06 | 0.86 | 0.00E+00 |
| H (freeze) | 1 x 6 | Transcription factors NAC | 8 | *Vitvi08g01264* | 1.63 | 1.37 | 0.94 | 0.00E+00 |
| H (freeze) | 1 x 6 | Jasmonate signaling | 9 | *Vitvi02g01182* | 2.70 | 1.28 | 0.81 | 3.77E-15 |
| H (freeze) | 1 x 6 | Glutathione metabolism | 10 | *Vitvi17g00395* | 3.29 | 1.34 | 0.83 | 1.11E-16 |
| I (freeze) | 1 x 15 | Transcription factors BZIP | 1 | *Vitvi16g01022* | 0.77 | 1.02 | 0.46 | 1.13E-04 |
| I (freeze) | 3 x 14 | Transferring nitrogenous enzymes | 2 | *Vitvi14g01808* | 0.87 | 1.05 | 0.46 | 1.41E-04 |
| I (freeze) | 2 x 11 | Transcription factors (bZIP) | 3 | *Vitvi08g01587* | 1.41 | 0.93 | 0.63 | 4.83E-08 |
| I (freeze) | 1 x 15 | Wnk family kinases | 4 | *Vitvi05g01833* | 3.03 | 1.36 | 0.69 | 9.85E-10 |
| I (freeze) | 1 x 14 | Electrochemical transporters | 5 | *Vitvi18g00480* | 2.10 | 1.16 | 0.62 | 8.88E-08 |
| I (freeze) | 1 x 12 | Orphans zf−b box | 6 | *Vitvi14g02476* | 1.24 | 0.95 | 0.73 | 2.13E-11 |
| I (freeze) | 1 x 15 | Tyrosine metabolism | 7 | *Vitvi18g02398* | 2.04 | 1.45 | 0.67 | 2.39E-09 |
| I (freeze) | 1 x 11 | Arginine and proline metabolism | 8 | *Vitvi06g01601* | 0.79 | 0.80 | 0.60 | 2.46E-07 |
| I (freeze) | 4 x 12 | ABA signaling | 9 | *Vitvi13g01623* | 1.63 | 0.85 | 0.65 | 1.43E-08 |
| I (freeze) | 1 x 14 | Transporters 66 to 94 | 10 | *Vitvi05g00342* | 1.29 | 1.02 | 0.69 | 8.58E-10 |
| J (accfreeze) | 1 x 38 | Starch and sucrose metabolism | 1 | *Vitvi04g01368* | 4.01 | 2.08 | 0.68 | 1.27E-09 |
| J (accfreeze) | 1 x 40 | Transcription factors (Helix−turn−helix) | 2 | *Vitvi18g00087* | 1.68 | 1.67 | 0.73 | 3.81E-11 |
| J (accfreeze) | 1 x 40 | Cytokinin signaling | 3 | *Vitvi16g00733* | 1.73 | 1.88 | 0.84 | 1.11E-16 |
| J (accfreeze) | 1 x 40 | Flower development | 4 | *Vitvi12g02353* | 5.43 | 2.51 | 0.80 | 1.83E-14 |
| J (accfreeze) | 1 x 37 | Glycosyltransferases | 5 | *Vitvi19g00255* | 3.64 | 1.29 | 0.58 | 9.07E-07 |
| J (accfreeze) | 1 x 38 | Starch and sucrose metabolism | 6 | *Vitvi10g00647* | 2.84 | 1.12 | 0.77 | 8.31E-13 |
| J (accfreeze) | 1 x 40 | Circadian rhythm | 7 | *Vitvi14g01469* | 2.49 | 1.47 | 0.45 | 1.77E-04 |
| J (accfreeze) | 1 x 38 | Chaperone HSP20 | 8 | *Vitvi18g00122* | 0.66 | 0.86 | 0.69 | 8.97E-10 |
| J (accfreeze) | 1 x 39 | Circadian rhythm | 9 | *Vitvi05g00071* | 4.74 | 1.53 | 0.69 | 7.52E-10 |
| J (accfreeze) | 1 x 36 | Tyrosine metabolism  (11. Thiamine metabolism) | 10 | *Vitvi10g00649* | 1.93 | 0.95 | 0.65 | 1.37E-08 |
| K (freeze) | 15 x 40 | Membrane ABC transporters | 1 | *Vitvi13g02110* | 1.40 | 1.53 | 0.46 | 1.08E-04 |
| K (freeze) | 13 x 40 | Amino-sugars metabolism | 2 | *Vitvi00g01651* | 3.44 | 2.15 | 0.89 | 0.00E+00 |
| K (freeze) | 13 x 40 | Glycosylases | 3 | *Vitvi10g02090* | 3.44 | 2.15 | 0.89 | 0.00E+00 |
| K (freeze) | 14 x 40 | Zeatin biosynthesis | 4 | *Vitvi08g02122* | 0.84 | 1.10 | 0.45 | 1.71E-04 |
| K (freeze) | 15 x 40 | Purine metabolism | 5 | *Vitvi05g00566* | 1.10 | 0.83 | 0.51 | 1.66E-05 |
| K (freeze) | 12 x 40 | Zeatin biosynthesis | 6 | *Vitvi10g02094* | 1.60 | 1.15 | 0.57 | 1.33E-06 |
| K (freeze) | 12 x 40 | Starch and sucrose metabolism | 7 | *Vitvi00g01655* | 1.60 | 1.15 | 0.57 | 1.34E-06 |
| K (freeze) | 12 x 40 | Acting on a peroxide as acceptor | 8 | *Vitvi07g02092* | 0.97 | 1.11 | 0.45 | 2.06E-04 |
| K (freeze) | 12 x 40 | ABC transporters | 9 | *Vitvi11g01488* | 1.39 | 0.91 | 0.70 | 3.06E-10 |
| K (freeze) | 13 x 40 | Transporters 66 to 94 | 10 | *Vitvi04g01969* | 1.39 | 0.96 | 0.58 | 5.73E-07 |

**Supplementary Table S2.** Description of genes coding for epigenetic factors located in spots A-K.

| **Spot** | **Gene ID** | **Gene description** | **BRITE category** | **Subcategory** |
| --- | --- | --- | --- | --- |
| **A** | *Vitvi05g00574* | protein argonaute 10 | Gene silencing | RISC (RNA-induced silencing complex) |
|  | *Vitvi08g00030* | uncharacterized | Chromatin remodeling factors | GBAF (ncBAF) complex |
| **B** | *Vitvi16g00174* | DNA (cyt-5)-methyltransferase CMT2 | Heterochromatin formation | Other proteins |
|  | *Vitvi12g00448* | protein argonaute 16 | Gene silencing | RISC (RNA-induced silencing complex) |
|  | *Vitvi08g00884* | protein argonaute 4A | Gene silencing | RISC (RNA-induced silencing complex) |
|  | *Vitvi11g00408* | protein argonaute PNH1 | Gene silencing | RISC (RNA-induced silencing complex) |
|  | *Vitvi06g01169* | uncharacterized | Gene silencing | RNA interference (RNAi) - Other proteins |
|  | *Vitvi08g01423* | WD repeat-containing protein LWD1 | Histone modification proteins | Polycomb repressive complex (PRC) and assoc. proteins - Noncanonical PRC1 (PRC1.3/1.5) |
| **C** | *Vitvi04g01275* | ATP-dependent DNA helicase DDM1 | Chromatin remodeling factors | Other chromatin remodeling factors |
|  | *Vitvi08g01666* | condensin complex subunit 1 | Chromosome condensation | Condensin I |
|  | *Vitvi12g00406* | condensin complex subunit 2 | Chromosome condensation | Condensin I |
|  | *Vitvi16g00486* | condensin complex subunit 3 | Chromosome condensation | Condensin I |
|  | *Vitvi05g00281* | condensin-2 complex subunit D3 | Chromosome condensation | Condensin II |
|  | *Vitvi05g00042* | condensin-2 complex subunit H2 | Chromosome condensation | Condensin II |
|  | *Vitvi06g00102* | DNA (cyt-5)-methyltransferase CMT3 | Heterochromatin formation | Other heterochromatin formation proteins |
|  | *Vitvi13g01699* | DNA topoisomerase 2 | Chromosome condensation | Other regulators |
|  | *Vitvi10g01549* | high mobility group B protein 7 | Nucleosome assembly factors | HMG (high mobility group) proteins |
|  | *Vitvi07g02212* | histone H1 | Nucleosome assembly factors | Histones |
|  | *Vitvi06g00423* | histone H2A | Nucleosome assembly factors | Histones |
|  | *Vitvi07g02097* | histone H2A variant 1 | Nucleosome assembly factors | Histones |
|  | *Vitvi14g00189* | histone H2A.1 | Nucleosome assembly factors | Histones |
|  | *Vitvi07g00219* | histone H2AX | Nucleosome assembly factors | Histones |
|  | *Vitvi13g00706* | histone H2B.3 | Nucleosome assembly factors | Histones |
|  | *Vitvi04g02223* | histone H3-like | Nucleosome assembly factors | Histones |
|  | *Vitvi13g01768* | histone H3.2 | Nucleosome assembly factors | Histones |
|  | *Vitvi06g00178* | histone H3.2 | Nucleosome assembly factors | Histones |
|  | *Vitvi06g00175* | histone H3.2 | Nucleosome assembly factors | Histones |
|  | *Vitvi13g00217* | histone H4 | Nucleosome assembly factors | Histones |
|  | *Vitvi13g01964* | histone H4 | Nucleosome assembly factors | Histones |
|  | *Vitvi06g00433* | histone H4 | Nucleosome assembly factors | Histones |
|  | *Vitvi06g00426* | probable histone H2B.1-like | Nucleosome assembly factors | Histones |
|  | *Vitvi19g00021* | protein Jade-1 | Histone modification proteins | HAT complexes - NuA3 complex |
|  | *Vitvi08g01767* | DNA (cyt-5)-methyltransferase CMT1 | Heterochromatin formation | Other heterochromatin formation proteins |
|  | *Vitvi16g00627* | structural maintenance of chromosomes protein 2-1 | Chromosome condensation | Condensin I |
|  | *Vitvi14g01953* | structural maintenance of chromosomes protein 4 | Chromosome condensation | Condensin I |
|  | *Vitvi13g01965* | uncharacterized | Nucleosome assembly factors | Histones |
|  | *Vitvi11g00622* | WD repeat-containing protein 76 | Heterochromatin formation | Other heterochromatin formation proteins |
| **F** | *Vitvi18g00684* | arginine N-methyltransferase 2 | Histone modification proteins | HMTs (histone methyltransferases) - PRMTs (protein arginine metyltransferases) |
|  | *Vitvi08g01185* | transcription factor-like protein DPA | Histone modification proteins | Polycomb repressive complex (PRC) and assoc. proteins - Noncanonical PRC1 (PRC1.6) |
| **G** | *Vitvi14g00050* | FACT complex subunit SPT16 | Nucleosome assembly factors | Histone chaperones - FACT complex |
| **J** | *Vitvi15g00864* | endoribonuclease Dicer homolog 1 | Gene silencing | RISC (RNA-induced silencing complex) |
|  | *Vitvi08g02244* | high mobility group B protein 1 | Nucleosome assembly factors | HMG (high mobility group) proteins |
|  | *Vitvi13g00031* | high mobility group B protein 1-like | Nucleosome assembly factors | HMG (high mobility group) proteins |
|  | *Vitvi15g00611* | probable helicase CHR10 | Chromatin remodeling factors | Other chromatin remodeling factors |
|  | *Vitvi11g00426* | arginine N-methyltransferase PRMT10 | Histone modification proteins | HMTs (histone methyltransferases) - PRMTs (protein arginine metyltransferases) |
| **K** | *Vitvi02g00549* | arginine N-methyltransferase 1.6 | Histone modification proteins | HMTs (histone methyltransferases) - PRMTs (protein arginine metyltransferases) |
|  | *Vitvi16g01407* | uncharacterized | Heterochromatin formation | Other heterochromatin formation proteins |

**Supplementary Image S3.** Principal component analysis of samples

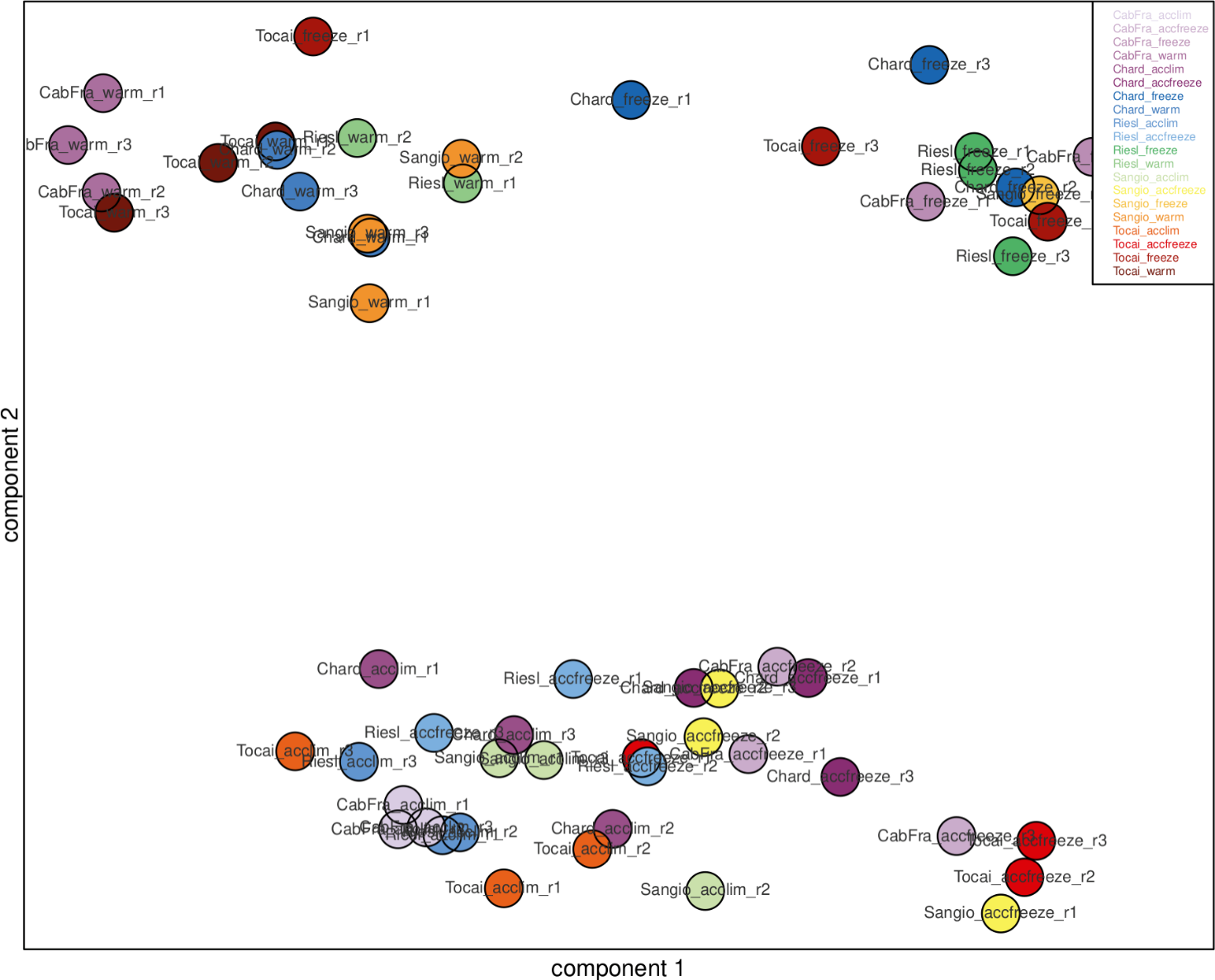

Supplement: Supplementary file 1 [file DataSheet_1.docx]
